# 1239. Early Lessons Learned: Implementation of a Statewide Mobile Application for Antimicrobial Stewardship in Colorado Using the PRISM Model

**DOI:** 10.1093/ofid/ofad500.1079

**Published:** 2023-11-27

**Authors:** Joana Dimo, Matthew J Weber, Christine MacBrayne, Matthew Miller, Sarah K Parker, Leigh Anne Bakel

**Affiliations:** University of Colorado/Children's Hospital Colorado, Denver, Colorado; University of Colorado/Children's Hospital Colorado, Denver, Colorado; Children's Hospital Colorado, Aurora, Colorado; Children's Hospital Colorado, Aurora, Colorado; University of Colorado/Children's Hospital Colorado, Denver, Colorado; University of Colorado/Children's Hospital Colorado, Denver, Colorado

## Abstract

**Background:**

Antimicrobial resistance, driven by improper antimicrobial use, is a global epidemic recognized as an urgent health threat by the Centers for Disease Control and Prevention (CDC). Previous collaborative work uncovered several self-identified barriers to antimicrobial stewardship in various Colorado hospitals (n=100), the most significant being lack of clinical guidelines, dosing knowledge in pediatric populations, and understanding stewardship methodologies.

**Methods:**

The overarching goal was to mitigate the self-identified barriers from prior studies through implementation of a mobile application decision support system (Firstline). We used the Practical Implementation Sustainability Model (PRISM) and RE-AIM (Reach, Effectiveness, Adoption, Implementation, and Maintenance) framework for implementation. We describe barriers encountered by our team as lessons learned for others considering similar implementation.

PRISM Domains and RE-AIM Framework

**Figure:**
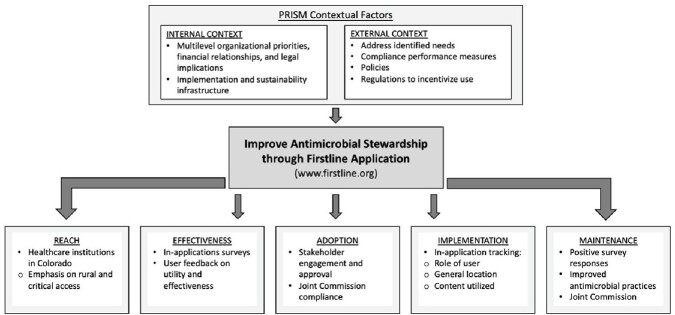

We used the Practical Implementation Sustainability Model (PRISM) to identify contextual factors and the RE-AIM (Reach, Effectiveness, Adoption, Implementation, and Maintenance) framework for implementation of a mobile application to improve antimicrobial stewardship in Colorado.

**Results:**

Utilizing PRISM domains, we evaluated if Firstline aligned with our organization’s priorities and offered an acceptable balance of practice and efficiency. Several barriers were encountered including navigating differing strategic planning approaches, financial relationships, and contracural arrangements among potential institutional partners, and navigating data sharing and liability for outfacing content. The external environment factors we evaluated included compliance performance measures, policies, and regulations to incentivize continued use of the application. We used the PRISM domains identified to develop our RE-AIM outcomes. Effectiveness will be measured through distribution of in-application surveys and user feedback. Implementation will be assessed though tracking use of the application by users, general location, and content used. For rural and critical access hospitals, adoption of the application may aid Joint Commission antimicrobial stewardship compliance, and is anticipated to contribute to maintenance.

**Conclusion:**

Use of PRISM helped elucidate and mitigate early implementation barriers including understanding institutional strategic planning, financial relationships, and legal significance of data sharing. Using RE-AIM, we will monitor our mobile application implementation success.

**Disclosures:**

**All Authors**: No reported disclosures

